# Prevalence of *Salmonella* in Eggs from Conventional and Cage-Free Egg Production Systems and the Role of Consumers in Reducing Household Contamination

**DOI:** 10.3390/foods12234300

**Published:** 2023-11-28

**Authors:** Doina Solís, Ninoska Cordero, Maritza Quezada-Reyes, Carla Escobar-Astete, Magaly Toro, Paola Navarrete, Angélica Reyes-Jara

**Affiliations:** 1 Laboratorio de Microbiología y Probióticos, Instituto de Nutrición y Tecnología de los Alimentos (INTA), Universidad de Chile, Santiago 8330015, Chile; doina.solis@inta.uchile.cl (D.S.); ncordero@inta.uchile.cl (N.C.); maritza.quezada@ug.uchile.cl (M.Q.-R.); carla.escobar@ug.uchile.cl (C.E.-A.); mtoroiba@umd.edu (M.T.); pnavarre@inta.uchile.cl (P.N.); 2 Joint Institute for Food Safety and Applied Nutrition (JIFSAN), University of Maryland, College Park, MD 20740, USA

**Keywords:** *Salmonella*, egg production, salmonellosis, antimicrobial resistance, public health

## Abstract

*Salmonella* is one of the leading causes of foodborne disease worldwide, usually related to contaminated poultry or poultry products, such as eggs. Since egg contamination with *Salmonella* depends on multiple factors that make it challenging to control, consumers’ knowledge about food safety and the proper handling of eggs is crucial. The aims of the study were (1) to determine the prevalence of *Salmonella* in eggs from conventional and alternative production systems, (2) to characterize the *Salmonella* isolates according to phenotypic-genotypic and antimicrobial-resistant traits, and (3) to understand how consumers manage the hazards related to egg contamination in the household. A total of 426 egg samples were analyzed (conventional systems = 240; alternative systems = 186). Culture-based and molecular microbiological methods were used to identify *Salmonella* and bioinformatics analysis of whole genome sequences was used to determine the serotype and antimicrobial-resistant genes. *Salmonella enterica* serotype Enteritidis was detected only in eggs from alternative systems (1.1%, 2/186). Isolates showed resistance to nalidixic acid (100%, 2/2), and the *aac(6′)-Iaa* gene and a mutation in the *gyrA* gene were identified in both isolates. Overall, consumers demonstrated knowledge regarding food safety; however, many still engage in practices that pose a risk of acquiring foodborne illnesses.

## 1. Introduction

Foodborne illnesses are considered one of the most important public health concerns worldwide. Millions of cases and related chronic health complications are documented yearly [1]. According to the World Health Organization (WHO), almost one in 10 people fall ill each year after eating contaminated food. That represents around 600 million cases and 420,000 deaths. Also, foodborne diseases significantly impact national economies, tourism, and trade [2].

Globally, one of the most frequently documented causes of foodborne disease is non-typhoidal *Salmonella*. In the United States, it is the second most common cause of foodborne outbreaks, and around 20% of the illnesses caused by *Salmonella* are related to poultry and poultry products, such as eggs [3,4]. In Chile, egg consumption accounted for 10.7% of all foodborne outbreaks reported by the Ministry of Health in 2019, primarily linked to inappropriate food handling at home [5].

Non-typhoidal *Salmonella* usually causes uncomplicated gastroenteritis in humans and usually manifests with fever, chills, vomiting, and diarrhea. Nonetheless, the disease can be life-threatening for vulnerable groups, such as children, the elderly, and patients with compromised immunity [6]. 

Worldwide, the production and consumption of eggs are constantly increasing and between 1961 and 2021 egg production increased from 14 to 1633 million tons [7]. China is the world’s largest hen egg producer, followed by India, Indonesia, and the United States. Altogether, these countries produce more than 940 million tons of eggs. In Chile, for instance, national production contributed about 4.5 million tons of eggs during 2021 [7]. In the United States, egg consumption was projected to be around 277.5 eggs per person in 2022. By 2023, it was predicted to reach 288.6 eggs per person [8]. In Chile, the annual egg consumption per person in 2018 was estimated to be around 230 eggs [9].

Over the years, egg production systems have diversified, mainly due to the growing concern for animal welfare. The European Union banned the use of conventional battery cages in 2012. These changes lead to the implementation of alternative systems, such as cage-free systems, which enhance the welfare of hens by permitting them to freely express their natural behavior and make decisions based on their needs and desires [10]. Nonetheless, in the alternative systems, hens might still be exposed to environmental stressors, predators, pests, and negative behaviors, such as intra-species aggression [11,12,13]. By 2022, it was estimated that around 25% of United States flocks were kept in cage-free systems [14]

The presence of *Salmonella* in eggs obtained from conventional systems depends on different factors, including the country and sampling methodologies [3]. Overall, egg contamination from industrial systems has been reported to be 0.005% in the United States, 0.37% in Europe, and between 0.5% and 5.6% in China [3,15,16]. However, for other pathogens, such as *Campylobacter* spp., free-range nest box swabs from alternative systems have shown a greater prevalence than cage swabs from conventional systems. The current data regarding pathogen prevalence between conventional and alternative systems remain unclear [17]. Furthermore, antimicrobial-resistant *Salmonella* has been isolated from eggs and egg products, including multi-resistant bacterial strains against β-lactams, fluoroquinolones, and aminoglycosides [18]. 

Since eggs might be a source of foodborne pathogens, such as *Salmonella*, consumers should be aware of their risks [4]. Unfortunately, only some consumers are aware of the hazards of consuming or manipulating contaminated eggs [19]. Considering the preparation and management of egg-containing foods at home, several factors might lead to salmonellosis, such as the natural contamination of eggs, consumers’ lack of knowledge regarding food safety, cross-contamination, and the deliberate consumption of raw or undercooked eggs. For instance, 25% of foodborne outbreaks are related to poor hygiene practices, such as cross-contamination [3,20,21]. 

The objectives of this study were: (1) to determine the prevalence of *Salmonella* in eggs from conventional and alternative production systems, (2) to characterize the *Salmonella* isolates according to phenotypic-genotypic and antimicrobial resistant traits, and (3) to understand how Chilean consumers manage the hazards related to egg contamination in the household.

## 2. Materials and Methods

### 2.1. Egg Samples

A total of 426 samples of eggs from conventional (battery-cage production systems) (*n* = 240) and cage-free (*n* = 186) systems were analyzed. The sample size was determined according to the results obtained with R software (Version 4.1.0) [22], based on the greater estimated prevalence of *Salmonella* in eggs reported for Europe for the last five years and the historical prevalence reported in the literature for alternative systems [23,24]. The number of samples analyzed for each production system exceeded the minimum required. Following the suggestions established by Public Health England, each sample consisted of six eggs for a total of 2556 eggs analyzed [25]. Samples were obtained from large (≥500,000 laying hens), medium (between 100,000 and 500,000 laying hens), and small (≤100,000 laying hens) egg producers [26], transported to the laboratory in their original package and analyzed within the same day. Sampling was conducted between May 2022 and January 2023.

### 2.2. Isolation and Identification of Salmonella

Microbiological analyses were carried out for eggshells according to the ISO 6579-1:2017 methodology [27]. For the pre-enrichment step, intact eggs were placed inside a sterile plastic bag containing 100 mL of preheated (37 °C) buffered peptone water (BD Difco^TM^, 218105, Franklin Lakes, NJ, USA) and gently rubbed for one minute. The eggs were removed from the bag in an aseptic way, and the bag was incubated at 37 °C for 24 h. Then, for the enrichment step, 0.1 mL of the pre-enrichments were inoculated onto Rappaport-Vassiliadis broth (Oxoid^TM^, CM0669, Waltham, MA, USA) and incubated at 42 °C for 24 h and 1 mL was inoculated in tetrathionate broth (Oxoid^TM^, CM0671, MA, USA) and incubated at 37 °C for 24 h. Ten microliters of the enrichments were plated onto Hektoen agar (BD Difco^TM^, 285340, NJ, USA) and Xylose Lysine Desoxycholate agar (XLD) (BD Difco^TM^, 278820, NJ, USA) and incubated at 37 °C for 24 h. At least three presumptive and atypical *Salmonella* colonies were analyzed and later confirmed through biochemical tests and polymerase chain reaction (PCR) for the *inv*A gene [28]. *Salmonella* isolates were stored at −20 °C for further analysis. 

### 2.3. Antibiotic Susceptibility Testing

Confirmed *Salmonella* isolates underwent antibiotic susceptibility testing using the Kirby–Bauer disk diffusion method for the following antibiotics: nalidixic acid (NAL = 30 µg), amoxicillin/clavulanate (AMC = 30 µg), amikacin (AMK = 30 µg), ampicillin (AMP = 10 µg), azithromycin (AZM = 15 µg), cefoxitin (FOX = 30 µg), ceftriaxone (CRO = 30 µg), chloramphenicol (C = 30 µg), ciprofloxacin (CIP = 5 µg), streptomycin (S = 10 µg), gentamicin (GE = 10 µg), imipenem (IMP = 10 µg), kanamycin (K = 30 µg), meropenem (MEM = 10 µg), tetracycline (TE = 30 µg), sulfonamide (S3 = 300 µg), trimethoprim-sulfamethoxazole (SXT = 1.25/23.75 µg), and florfenicol (FFC= 30 µg). Results were interpreted according to Clinical and Laboratory Standards Institute (CLSI) guidelines [29]. *E. coli* ATCC 25922 and *Staphylococcus aureus* ATCC 25923 were used as control strains (CLSI, 2020). 

### 2.4. Salmonella Whole Genome Sequencing (WGS) Analysis

Overnight cultures of the two *Salmonella* isolates grown in tryptic soy broth (BD Difco^TM^ 22092, USA) were used for the genomic DNA extraction using the DNeasy Blood and tissue kit (Qiagen, Germantown, TN, USA) following the protocol for Gram-negative bacteria recommended by the manufacturer. Libraries were prepared with the Illumina DNA pep library preparation kit (Illumina Inc., San Diego, CA, USA). Paired-end sequencing (2 × 150 bp) was performed in a NextSeq instrument using the Illumina NextSeq Reagent Kit P2 (300 cycles) according to the manufacturer’s instructions. 

The sequenced data was deposited in the National Center for Biotechnology Information (NCBI) Sequence Read Archive (SRA) under numbers SRR26050061 and SRR26050062. Read quality control, read trimming, de novo assembly, and assembly quality control were performed in the GalaxyTrakr platform [30] using the FastQC [31], Trimmomatic v0.36.4 [32], Spades v3.12.0 [33], and QUAST v5.0.2 [34] tools. Sequence type (ST) determination and serovar prediction were determined using the MLST 2.22.0 [35] and sistr_cmd [36] in GalaxyTrakr tools.

Phylogenetic relatedness among *Salmonella* isolates was analyzed through a single nucleotide polymorphism (SNP) calling in the CSI Phylogeny 1.4 from the Center for Genomic Epidemiology (CGE) at Denmark Technical University (https://cge.food.dtu.dk/services/CSIPhylogeny/, accessed on 12 September 2023) with default parameters [37]. *Salmonella* serotype Enteritidis strain 92-0392 (CP018657.1) was used as the reference genome, and a difference of <20 SNPs was used to infer genetic relatedness between the isolates [38]. Finally, the presence of antimicrobial resistance genes was determined with AMRFinder, also from CGE [39].

### 2.5. Survey Implementation 

An online survey to analyze the consumers’ knowledge and practices regarding food handling at home was prepared according to previously published surveys [3,19,21,40,41,42,43]. A sample size of 385 respondents was calculated according to the sample size formula for infinite populations [44]. The survey sample was non-probabilistic based on quotas according to the age group (18–29, 30–59, and 60+) (Supplementary Materials S1).

Closed (*n* = 19) and multiple-choice (*n* = 16) questions were included, and responses were received from 17 January through 28 February 2023. The survey gathered personal and demographic data, consumer habits regarding the purchase, storage, handling, and consumption of eggs, and food safety knowledge. The study was approved by the Institute of Food Nutrition and Technology (INTA) Scientific and Ethical Committee (CEC) from the University of Chile (Number 004/2023). Each participant agreed to answer the survey by accepting the informed consent form. 

### 2.6. Statistical Analysis

Fisher’s exact test was used to compare the prevalence of *Salmonella* depending on the origin of the analyzed eggs (conventional vs. alternative production system). A *p*-value of ≤0.05 was considered significant. Descriptive and inferential statistics using SPSS Statistics Software (IBM, version 21) were used to analyze the survey’s answers. The Chi-square test was employed to determine the relationship between age groups and the survey answer [45]. Cramer’s V test was used to estimate the intensity or strength of the association, where the significance of variables according to the Chi-square test was determined at a *p*-value < 0.05. According to Akoglu (2018), the interpretation of the Cramer’s V test strength of the association was categorized as very strong (>0.25), strong (0.15–0.25), moderate (0.10–0.14), weak (0.05–0.09), and no or very weak (0–0.04) [46,47]. Adjusted standardized residuals (ASR) were used to estimate the magnitude and directionality of the relationship of the differences. The ASRs were considered statistically significant when the value was greater than 1.96 or less than −1.96 [48].

## 3. Results

### 3.1. Prevalence of Salmonella in Eggs from Different Production Systems 

**Figure 1:**
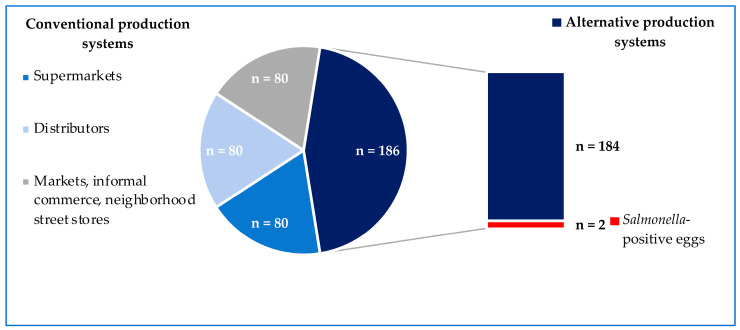
Prevalence of Salmonella according to the egg-production system.

Salmonella was not detected in eggs from conventional battery-cage systems (0/240), while 1.1% (2/186) of samples from alternative cage-free systems carried Salmonella (Figure 1). Samples carrying Salmonella came from different batches from the same egg producer and were obtained eight months apart. No statistical differences were found between the Salmonella prevalence from conventional and alternative egg production systems (*p*-value = 0.1). 

### 3.2. Phenotypic and Genomic Characterization of Salmonella Isolates

One *Salmonella* isolate was selected from each positive sample for further characterization, which was analyzed according to the Kirby–Bauer disc diffusion method and showed 100% (2/2) resistance to nalidixic acid while being susceptible to the remaining antibiotics tested. The bioinformatic analysis predicted that both isolates were *Salmonella enterica* subsp. *enterica* serotype Enteritidis (*S*. serotype Enteritidis), ST-11. Both genomes carried the *aac(6′)-Iaa* gene, which confers resistance to tobramycin and amikacin, and a mutation of the *gyrA* gene (TCC to TTC; Asp83Gly), conferring resistance to nalidixic acid. Nineteen SNPs were identified between both isolates, indicating a close phylogenetic relatedness, although the isolates were not clones [38,49].

### 3.3. Home Egg Management and Food Safety

A total of 385 answers were obtained from the online survey. A summary of the most relevant demographic data from the consumers is shown in Table S1. The survey was mainly answered by middle-aged (53.2%, 205/385) women (82.6%, 318/385). From the survey participants, 17% (65/385) declared to be related to the food handling and/or food safety field (Table S1). Table 1 shows the most representative responses regarding consumers’ habitual egg-purchasing behavior according to age, particularly the distinction between conventional and alternative egg production systems. Results showed that 40.8% of respondents prefer eggs from alternative systems, while 46.8% indicated that the production system was irrelevant to their purchasing decision. 

The survey results revealed notable trends in household egg-handling practices (Table 2). Around 20% of respondents admitted buying cracked or broken eggs, a practice associated with potential food safety risks. The analysis revealed that this practice was less frequent in older adults than in other age groups (Cramer’s V test = 0.164). In addition, almost a quarter (24.9%) of respondents reported washing eggs before storing them, despite the widespread advice against such practice due to the risk of introducing contaminants through the pores of the eggshell (Table 2). 

The responses were strongly associated with middle-aged consumers who demonstrated safer hygiene practices (Cramer’s V test = 0.150), such as not washing eggs before storage. Furthermore, over half of the respondents showed awareness of proper egg storage practices, with the vast majority choosing to store eggs in the refrigerator (68.5%) (Table 3).

Regarding preferences in egg consumption (Table 4), it is noteworthy that 46.3% of adults over 60 years old reported consuming raw or undercooked eggs in home-cooked dishes. Interestingly, within this demographic group, 93.9% manifested confidence that the proper cooking of eggs could prevent the onset of foodborne disease. 

Consumers’ perception of food safety concerning different eating places and egg products was assessed. Most participants (76.5%) believe eating out increases the likelihood of becoming ill, particularly street food. In contrast, the prevailing consensus among respondents was that homemade food has minimal food safety risks (Table 5).

## 4. Discussion

Poultry and poultry products have been identified as major sources of foodborne diseases and pathogen transmission [50,51], and *S*. Enterica is the primary pathogen related to eggs. The industry makes efforts to improve egg safety and lower the *Salmonella* prevalence through good manufacturing practices (GMP) and hazard analysis and critical control points (HACCP) systems. However, as bacteria keep genetically evolving, it is becoming more challenging to control this pathogen. 

Egg production systems have evolved over the years adding another significant food safety challenge [50]. Due to political, commercial, and social pressure, egg production systems have changed from conventional caged systems to alternative methods, like cage-free or free-range, particularly in regions such as the United States and Europe [50]. Animal welfare is an important aspect that should be considered in every production system; nevertheless, since many food safety measures were designed for conventional production systems, which have a higher level of control, their efficacy might not be the same for alternative systems [50,51]. 

In the United States, for example, through the Egg Safety Final Rule issued in 2009, the Food and Drug Administration (FDA) highlighted essential measures to prevent the on-farm infection of hens with *Salmonella*, the potential production of contaminated eggs, and the proper actions that should be considered after laying eggs [52]. Nonetheless, due to different factors, such as the exposure of hens to vectors, these measures are incompatible with alternative systems that allow hens to roam outdoors [53]. 

As a result, in 2013 the FDA provided guidance for layers with access to areas outside the poultry house. However, the guidance document emphasizes that the provided information should be considered merely as a suggestion unless explicit regulatory or statutory obligations are referenced. Finally, only farms with 3000 or more laying hens are subjected to the Egg Rule [52,54].

In this study, we did not observe significant differences between the presence of *Salmonella* in conventional or alternative production systems (*p* = 0.1). However, we detected that two samples (1.1%, 2/186) obtained from cage-free hens that roam in open, less protected environments were contaminated with *Salmonella* serotype Enteritidis. These conditions could increase the likelihood of *Salmonella* contamination in egg production environments. Although the following factors were not analyzed in the study, egg safety, and thus the presence of *Salmonella* in eggs obtained from alternative systems, also depends on the flock density, the exposure to contaminated feces and substrate, dust, vectors, the stress level of the hens, and the location of the nesting boxes, among others. It must be considered that the internal content of eggs might also be contaminated and present different levels of *Salmonella* compared to the egg’s surface; therefore, the reported results can be underestimated.

Even though eggs can be contaminated on the surface and internally, the present study sought to determine the prevalence of *Salmonella* from eggshells due to the close relationship between eggshell contamination and the risk of cross-contamination during egg handling by consumers. In U.S.-based studies, for instance, no differences were reported in the contamination of egg contents with *Salmonella* between conventional and alternative egg production systems, while differences have been reported with respect to the prevalence of Salmonella in droppings related to the surface contamination of eggs [50].

The prevalence of *Salmonella* from the egg’s internal contents might be influenced by the measures implemented in the poultry industry, where control is based on a combination of strategies such as the purchase of replacement birds from free breeders, proper feed, biosecurity measures, management of gut microbiota, and vaccination [55]. Moreover, the contamination of the internal contents of eggs is considered a rare finding [56]. 

Although *Salmonella* was only related to alternative production systems, globally, data on the impact of different production systems on pathogen prevalence remains elusive [17,50,57]. Therefore, the way alternative systems are implemented raises concerns about the presence of pathogens and measures that should be applied. 

To promote animal welfare, enriched cage systems might be a balanced alternative over conventional production systems since they combine the laying productivity of hens and improved animal welfare. These systems provide perches, scratch pads, nest boxes, and larger areas, which offer an improved environment over conventional cages [57,58].

In the United States, for instance, efforts have been made to establish more detailed measures to ensure the welfare of laying hens. As stated by the USDA, the U.S. egg industry has increasingly changed from conventional battery-caged systems to cage-free production, nearly doubling it in the 5-year span of 2017 to 2021 [59].

The Chilean Agricultural and Livestock Service created guidelines where important recommendations were established for both conventional and alternative systems to promote animal welfare. These guidelines promote welfare aspects regarding water and diet, health measures, housing, behavior, laying productivity, productive management practices, and training and education of welfare managers and staff [26].

*Salmonella* serotype Enteritidis is the predominant serovar found in eggs and has been linked to outbreaks worldwide. Recently, a study reported the involvement of *Salmonella* serotype Enteritidis ST11 in an outbreak of salmonellosis traced to a restaurant where eggs and chicken were considered the most likely cause of the outbreak [60]. This incident highlights the importance of solid control measures in egg handling and production. Vigilant monitoring, stringent hygiene practices, and strict temperature control throughout the egg supply chain are essential to reduce the risk of *Salmonella* contamination and protect public health.

Antibiotics in the poultry industry and other animal production systems are used for therapeutic and preventive control of diseases and to improve feed efficiency and productivity. However, their improper use might result in the dangerous accumulation of these chemicals in animal tissues and eggs and promote the selection of resistant bacteria [61]. The main concern regarding antimicrobial resistance is the failure to treat animal and human infections with already available antibiotics, thus reducing the chances of using complementary therapies, such as chemotherapy and surgery [62]. In this study, both *S.* serotype Enteritidis isolates were resistant to nalidixic acid, which has been previously reported in *Salmonella* isolated from eggs [18,63]. In *Salmonella,* quinolone resistance is usually related to mutations in the quinolone resistance determining region of the *gyrA* and *parC* genes [64,65]. Vuthy et al. (2017) described that over 75% of nalidixic acid-resistant *Salmonella* isolates had a mutation in the *gyrA* gene. On the other hand, our identified isolates were not phenotypically resistant to aminoglycoside amikacin [65]. Still, the bioinformatics analysis identified the gene *aac(6′)-Iaa* that confers resistance to tobramycin, amikacin, and kanamycin [66,67]. As shown in our results, it has been reported that the presence of the *aac(6′)-Iaa* gene is not necessarily related to phenotypic resistance to aminoglycosides since this gene is often weakly expressed or not expressed at all [68].

Survey results showed that consumers from every age range preferred alternative egg production systems (40.8%, 157/385) over conventional production systems (3.9%, 15/385). Nonetheless, no statistical relationship was demonstrated between this preference and the age group (*p* > 0.05) (Table 1). This is a significant result, considering that most eggs around the world are still produced in conventional systems and that alternative egg production systems in Chile only account for 1.2% of the available production systems [9,69]. Worldwide, as shown by Sinclair et al. (2022), consumers prefer to buy eggs from hens that have not been kept in cages [69].

Due to the complex nature of egg contamination with *Salmonella* and the multiple influencing factors contributing to its management, it remains critical to ensure that foods containing eggs from alternative production systems are handled and prepared appropriately. Our findings suggest that such systems are susceptible to *Salmonella* contamination, emphasizing the continued significance of such measures in preventing foodborne illnesses [51].

As stated by Cardoso et al. (2021) the first step for the safe handling of eggs the awareness of the potential biological dangers that might be present in eggs. To reduce the probability of acquiring salmonellosis from eggs, adequate practices must be focused on from the purchase to the consumption of egg-containing foods. Overall, consumers should examine eggs at the time of purchase, avoid washing the eggs before storage, and refrigerate the eggs. In addition, it is important to comply with the appropriate cooking temperature and time to eliminate *Salmonella* before consumption [3].

When analyzing consumer food handling habits at home, we observed that most consumers across all age groups avoid dangerous handling practices such as using cracked or dirty eggs (80.8%, 311/385), washing eggs before storage (75%, 289/385), preparing and consuming foods containing raw or undercooked eggs (59.5%, 229/385), among others; nevertheless, over 40% of the consumers do prepare and consume foods containing raw eggs (40.5%, 156/385) (Table 4).

It should be noted that *Salmonella*-associated foodborne outbreaks are mainly linked to contaminated eggs and raw egg products, such as sauces and dressings, like mayonnaise, hollandaise, aioli and egg butter mayonnaise, desserts such as tiramisu, mousse, and fried-ice cream batter, and drinks, such as raw egg protein drinks and eggnog [70,71]. Interestingly, regarding consumers’ knowledge about food safety, 96.6% (372/385) of respondents stated that proper cooking of eggs could prevent people from becoming ill (Table 4).

It is crucial to outline that although in some countries, such as the United States, all shell eggs and egg products packed for consumers must be refrigerated [72], in Chile, while the survey respondents preferred to refrigerate eggs, it is not a legal requirement [73], and the refrigeration of eggs at home is a consumer’s decision based on their food safety knowledge and practices.

In this survey, most respondents (76.5%, 254/385) think eating street food increases the likelihood of catching a foodborne illness, while only 4.8% (16/385) considered homemade food a risk. However, global evidence shows that most foodborne outbreaks typically occur in homes. Although results may vary among countries, Gargiulo et al. (2022) reported that household outbreaks accounted for 41.3% of all European cases in 2019, while restaurants, cafes, bars, and street food accounted for 28.6% [74].

One of the drawbacks of this research is that to determine the appropriate sample size for detecting *Salmonella* in eggs, we utilized European data rather than local information which was not available [23,24]. Furthermore, data on the prevalence of *Salmonella* from different egg production systems is not available in Chile. In addition, the recovery of *Salmonella* from eggs could increase with a bigger sample size. Nevertheless, this research produces publicly available data that can be utilized to develop studies with more precise estimations.

Overall, the safety of eggs and egg products remains a public health concern, particularly related to the contamination of *Salmonella* serotype Enteritidis. Our study showed the importance of addressing the challenges related to the shift from conventional egg production systems to alternative systems and the importance of understanding antimicrobial resistance mechanisms to develop tailored control approaches. Consumers’ education on food safety plays a crucial role in reducing foodborne illnesses since *Salmonella*-related disease is an ongoing threat that should be appropriately prevented. The cooperation and work between regulatory bodies and egg industry representatives must always prevail.

## 5. Future Perspective and Conclusions

Although the prevalence of *Salmonella* from eggs was low, this pathogen was only detected from alternative egg production systems. To our knowledge, this is the first Chilean study to compare the prevalence of *Salmonella* from conventional and alternative egg production systems. Efforts for reducing *Salmonella* are ongoing and should be adapted and improved for new alternative production systems since the effectiveness of existing egg safety measures developed for conventional production systems might not translate to the alternative ones. Responses to the survey, which complemented the *Salmonella* prevalence results, highlighted the need for increased food safety education and adequate food handling practices at home. The interplay between foodborne pathogens, the diversification of egg production systems, and consumer behavior represent challenging topics for ensuring food safety and culture. In addition, our findings suggest that more work should be done to prevent the selection of antibiotic-resistant strains arising from the inappropriate use of antibiotics in animal production systems, including poultry.

The future perspectives of this study include the comparison of the present results with the evaluation of the pathogens from the egg’s internal contents and determining the association between pathogen prevalence and the hygiene practices carried out by producers according to the production system.

## Figures and Tables

**Table 1:** Preference of the egg production system according to the age of the respondent.

Age of Respondent	Conventional Production System N (%)	Alternative Production System N (%)	Irrelevant N (%)	Do Not Know N (%)	χ^2^ *
18–29	2 (2)	38 (38.8)	49 (50)	9 (9.2)	0.257
30–59	6 (2.9)	84 (41)	99 (48.3)	16 (7.8)	
≥60	7 (8.5)	35 (42.7)	32 (39)	8 (9.8)	
Total	15 (3.9)	157 (40.8)	180 (46.8)	33(8.6)	

* Chi-square test for the association between the respondent’s age and the preferred production system.

**Table 2:** Most representative answers related to food handling habits at home and food safety knowledge of consumers.

Question	Age of Respondent	Yes N (%)	No N (%)	χ^2^ *	Cramer’s V Test **
Do you buy cracked or dirty eggs?	18–29	25 (25.5)	73 (74.5)	0.006	0.164
	30–59	43 (21)	162 (79)		
	≥60	6 (7.3) ^(−)^	76 (92.7) ^(+)^		
	Total	74 (19.2)	311 (80.8)		
Do you clean or wash the eggs before storage?	18–29	25 (25.5)	73 (74.5)	0.013	0.150
	30–59	41 (20) ^(−)^	164 (80) ^(+)^		
	≥60	30 (36.6) ^(+)^	52 (63.4) ^(−)^		
	Total	96 (24.9)	289 (75)		

* Chi-square test (*p*-value) for the association between the respondent’s age and the survey answer. ** Strength of the association according to Cramer’s V test. The significance level was established at *p* < 0.005 according to the Chi-Square test. (+) Adjusted standardized residuals (ASR) > 1.96 denotes that the variable was observed more frequently than expected; (−) Adjusted standardized residuals (ASR) < −1.96 denotes that the variable was observed less frequently than expected.

**Table 3:** Egg Storage preferences in households.

Age of Respondent	Refrigerator Door N (%)	Inside of the Refrigerator N (%)	Room Temperature N (%)	χ^2^ *
18–29	41 (41.8)	24 (24.5)	33 (33.7)	0.4
30–59	72 (35.1)	72 (35.1)	61 (29.8)	
≥60	28 (34.1)	27 (32.9)	27 (32.9)	
Total	141 (36.6)	123 (31.9)	121 (31.4)	

* Chi-square test (*p*-value) for the association between the respondent’s age and the survey answer.

**Table 4:** Consumer preferences and food safety awareness about egg consumption.

Question	Age of Respondent	Yes N (%)	χ^2^ *
Do you consume foods containing raw or undercooked eggs prepared at home?	18–29	40 (40.8)	0.433
	30–59	78 (38)	
	≥60	38 (46.3)	
	Total	156 (40.5)	
Do you think proper cooking of eggs could prevent foodborne illness?	18–29	95 (96.9)	N/A **
	30–59	200 (97.6)	
	≥60	77 (93.9)	
	Total	372 (96.6)	

* Chi-square test for the association between the respondent’s age and the survey answer. ** Not applicable.

**Table 5:** Places where consumers believe they are more likely to become ill after consuming raw or undercooked egg-containing foodstuffs.

Age of Respondent	Restaurants N (%)	Food Trucks N (%)	Street Food N (%)	Household N (%)	Other N (%)
18–29	5 (5.6)	6 (6.7)	72 (80.9)	6 (6.7)	0 (0)
30–59	15 (8.4)	16 (9)	137 (77)	9 (5.1)	1 (0.6)
≥60	11 (16.9)	7 (10.8)	45 (69.2)	1 (1.5)	1 (1.5)
Total	31 (9.3)	29 (8.7)	254 (76.5)	16 (4.8)	2 (0.6)

## Data Availability

Data is contained within the article or supplementary material.

## Supplementary Materials

The following supporting information can be downloaded at: https://www.mdpi.com/article/10.3390/foods12234300/s1, Table S1: Demographic characteristics of survey participants (*n* = 385). Supplementary Materials S1: Survey.
