# Supplementary material for: Prevalence of Salmonella in Eggs from Conventional and Cage-Free Egg Production Systems and the Role of Consumers in Reducing Household Contamination
Source: Foods. 2023 Nov 28;12(23):4300. doi: 10.3390/foods12234300 (PMC10706720; doi:10.3390/foods12234300)
Supplement: Supplementary file 1 [file foods-12-04300-s001.zip › foods-2701711-Supplementary Materials S1--Survey_Solis et al.pdf]

Egg handling practices at home and food safety knowledge of Chilean consumers

From our consideration,

Thanking you in advance for your interest in participating in this study, we inform you that the main purpose of this survey is to investigate the practices and knowledge of consumers in Chile regarding the handling of eggs at home. Participation is open to all Chilean residents over 18 years of age who **buy, prepare, and consume** eggs or egg products regularly in their homes. People who do not practice the three aspects mentioned above are excluded.

Your collaboration will be of great help in informing the population about the most important factors that affect food safety, particularly egg safety, considering the concept of safety as the absence of hazards in food that could harm the health of consumers. It should be emphasized that participation in this study is completely voluntary, is risk-free and can be withdrawn at any time without consequence.

If you wish to continue, please be informed that this survey has a total of 35 questions with an estimated duration of 9 to 10 minutes. All information you provide during this study will be kept strictly confidential and will only be used for research purposes and will not be available for other purposes. Your answers will be kept for no longer than 12 months under the responsibility of the researcher in charge. For your participation to be considered, please remember to answer all the questions (35), otherwise your answers will not be valid for the analysis.

Your identity will remain anonymous and there will be no financial compensation for your participation.

No answer is incorrect, we are only interested in your honesty. If you have any questions, comments, or suggestions about this survey, please contact Angélica Reyes Jara. Her telephone number is +56229781515 and her email address is areyes@inta.uchile.cl. If you have any questions or concerns about your rights as a participant in this study, you may contact Dr. Paulina Correa, Chair of the Ethics Committee of the Institute of Nutrition and Food Technology (INTA) at correo.etica@inta.uchile.cl.

Do you agree to participate in this survey?

- Yes
- No

Section 1: Personal and demographic data

1. **What gender do you identify with?**
- Female
- Male
- Other: _____

2. **What is your nationality?**
- Chilean
- Peruvian
- Colombian
- Haitian
- Venezuelan
- Bolivian
- Other: ____

3. **What age range are you in?**
- 18 to 29 years old
- 30 to 59 years old
- 60 years or more

4. **What is your area of residence?**
- Great North (XV, I, II)
- Norte Chico (III, IV)
- Central zone (V, RM, VI, VII, XVI)
- Southern Zone (VIII, IX, XIV, X)
- Southern zone (XI, Magallanes and Chilean Antarctica)

5. **Do you study or work in an area related to food handling?**
- Yes
- No

Section 2: Practices in the purchase (CCH1), storage, hygiene (CCH2 and CCH3), preparation and consumption of eggs (CCH4, CCH5 and CCH6).

PURCHASE

1. **Where do you buy your eggs? Check the option(s) that apply:**

- Supermarkets
- Neighborhood stores
- Markets such as Lo Valledor, Franklin, among others.
- Free fairs
- Internet with other providers
- Local producers in the field or backyard hen farms
- Other: ____

2. **For what reason do you shop at these locations? Check the option(s) that apply:**

- Hygiene
- Price
- Variety
- Proximity to place of residence
- Other: ____

3. **Regarding the type of egg production system, when buying or obtaining eggs, you are interested in:**

- Conventional battery-caged production
- Alternative or free-cage production
- I am indifferent
- Do not know

4. **Do you buy eggs that are cracked or dirty (with feces)?**

- Yes
- No

STORAGE

5. **Do you wash or clean the eggs before storing them?**
- Yes
- No

6. **Where do you store eggs in your home?**
- On the refrigerator door
- Inside the refrigerator
- At room temperature
- Other: ____

7. **When you store eggs, do you keep them in their original package (egg carton) or other container away from other foods?**
- Yes
- No

8. **Do you prepare foods containing raw eggs such as mayonnaise, sauces, or desserts?**
- Yes
- No

9. **If yes, do you refrigerate these products after preparation?**
- Yes
- No

FOOD PREPARATION

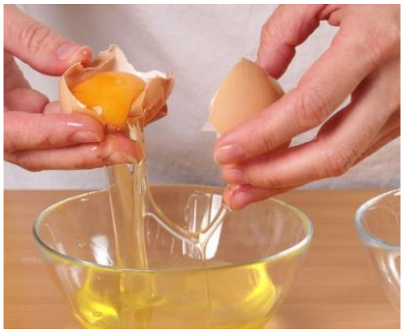

10. **Do you usually separate the yolks from the whites by passing the yolk from one shell to the other as shown in the picture?**

- Yes
- No, I use a yolk-white separator
- No, I do it with my hands
- I do not perform this action
- Other:___

11. **When you are handling an egg in your kitchen, do you keep it away from other foods?**

- Yes
- No

12. **When preparing eggs or foods containing eggs, where do you usually get the information to prepare them? Check the option(s) that apply:**

- o Internet
- o Cookbooks / magazines
- o Family and friends
- o Television programs
- o Product labels
- o I do not follow recipes
- o Other: ____

HYGIENE

13. **Do you wash your hands with soap and water or sanitizer after handling an egg?**

- Yes
- No

14. What do you do to clean cooking utensils that came in contact with the raw egg before reusing them to prepare another food?

- Use of water and detergent
- Use of water and chlorine
- Use only water
- I use a damp cloth
- I use a paper towel
- I do not clean them

15. What do you do to clean surfaces that came in contact with the raw egg before reuse?

- Use of water and detergent
- Use of water and chlorine
- Use of water and alcohol
- Use only water
- I use a damp cloth
- I use a paper towel
- I do not clean them

CONSUMPTION

16. On average, how long does it take to eat all the eggs the you purchase?

- 1 week
- 2 weeks
- 3 weeks
- More than 4 weeks
- I do not estimate the time

17. Do you often consume foods containing raw or undercooked eggs prepared at home? Such as: mayonnaise, sauces, desserts, soft-boiled eggs, etc.

- Yes
- No

18. **In your opinion which of the following images corresponds to a fried cooked egg?**

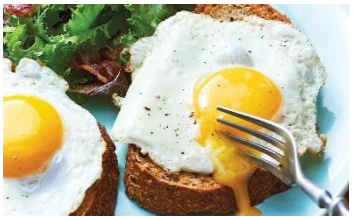

A

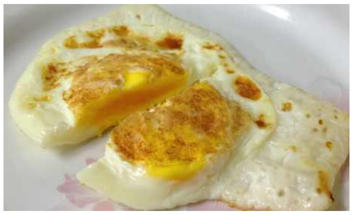

B

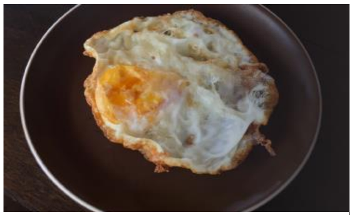

C

Section 3: knowledge about food safety applied in the consumption and hygiene in the handling of eggs at home

CONSUMPTION

1. **Do you believe that commercially branded eggs are safe to eat?**
   - Yes
   - No

2. **Do you believe that eggs purchased or obtained from backyard or free-range hens without a commercial brand are safe to eat?**

- Yes
- No

3. **Do you think there is any health risk in consuming raw, undercooked eggs or foods containing them?**

- Yes
- No

4. **If you believe there is a risk, where do you think you are most likely to get sick from eating a food made with raw or undercooked eggs?**

- Restaurants
- Food trucks or food carts

- Street food
- Home
- Other: ____

5. Which of the following microorganisms do you associate with the consumption of eggs?

- *Salmonella*
- *E.coli*
- *Listeria*
- *Campylobacter*
- None
- I do not know

6. Is it safe for older adults, children, pregnant women and immunocompromised people to consume raw or undercooked eggs or foods containing eggs?

- Yes
- No

7. Could proper cooking of eggs prevent illness?

- Yes
- No

HYGIENE

8. Do you think there is any risk in handling an egg without washing with soap and water or disinfecting your hands?

- Yes
- No

9. Do you think it is important to wash utensils that have been in contact with the egg before reusing them?

- Yes

- No

10. Do you think it is relevant to clean or disinfect the surfaces that were in contact with the egg before using them again?
  - Yes
  - No

11. Do you consider important to keep eggs away from other types of food both from where you store them and when preparing them?
- Yes
- No

End of the survey

1. Would you be willing to receive more information on the proper storage and handling of the eggs that you buy?
- Yes
- No

2. If you wish, you can write your email to receive more information:

____
