# Supplementary material for: Prevalence of Salmonella in Eggs from Conventional and Cage-Free Egg Production Systems and the Role of Consumers in Reducing Household Contamination
Source: Foods. 2023 Nov 28;12(23):4300. doi: 10.3390/foods12234300 (PMC10706720; doi:10.3390/foods12234300)
Supplement: Supplementary file 1 [file foods-12-04300-s001.zip › foods-2701711-supplementary-Table S1.pdf]

Supplementary Table

Table S1. Demographic characteristics of survey participants (n = 385).

Question	Answer	N (%)
**1. Gender**	Female	318 (82.6)
	Male	64 (16.6)
	Other	3 (0.8)
**2. Age**	18–29 years	98 (25.3)
	30–59 years	205 (53.2)
	≥ 60 years	82 (21.4)
**3. Do you study/work in the food handling/safety field?**	Yes	65 (16.9)
	No	320 (83.1)
